# 

**Published:** 1837-04

**Authors:** 


### BOOKS RECEIVED FOR REVIEW.

ENGLISH.

1. A Treatise on Consumption; embracing an Enquiry into the Influence exerted upon it by Journeys, Voyages, and Change of Climate, &c. Adapted for general Readers. By Wm. Sweetser, M.D.—Boston, 1836. 8vo. pp. 254.

2. Anatomical, Pathological, and Therapeutic Researches upon the Disease known by the name of Gastro-Enterite, Putrid, Adynamic, Ataxic, or Typhoid Fever, &c. By P. C. A. Louis, M.D. &c. Translated from the French, by H. I. Bowditch, M.D. — Boston, 1836. Two Vols. 8vo. pp. 395, 462.

3. Elements of the Practice of Medicine. By R. Bright, M.D., and T. Addison, M.D., Lecturers on the Practice of Medicine at Guy's Hospital. Part I.—London, 1836. pp. 128. 4s.

4. An Elementary System of Physiology. Complete in one Volume. By John Bostock, M.D. F.R.S. &c. Third Edition, revised and corrected throughout.—London, 1836. 8vo. pp. 887. 20s.

5. Medical Essays. By J. Hungerford Sealy, M.D.E. A.B. T.C.D. No. I. Phthisis Pulmonalis, its History and Varieties, &c. —London, 1837. 12mo. pp. 82.

6. Lectures illustrative of certain local Nervous Affections. By Sir Benjamin Brodie, Bart. F.R.S., Serjeant Surgeon to the King, &c.—London, 1837. 8vo. pp. 88.

7. Digest of the Homœopathic Principles. By E. W. Williams, M.B. Cantab.—London, 1837. 12mo. 2s.

8. Critical Remarks on certain recently published Opinions concerning Life and Mind. By John Roberton, Surgeon, &c., Manchester.—London, 1836. 8vo.

9. Practical Observations on Nervous and Sympathetic Palpitation of the Heart, &c. By J. C. Williams, M.D., Physician to the Nottingham Dispensary, &c.—London, 1836. 8vo. pp. 130.

10. Sketch of the Comparative Anatomy of the Nervous System; with Remarks on its Development in the Human Embryo. By John Anderson, M.E.S.—London, 1837. 4to. pp. 63; with Plates. 8s.

11. A Practical Treatise on Diseases of the Skin, arranged with a view to their Constitutional Causes and Local Characters. By Samuel Plumbe, Surgeon, &c. Fourth Edition, considerably enlarged, and with additional Engravings. — London, 1837. 8vo. pp. 607. 21s.

12. A Clinical Treatise on the Endemic Fevers of the West Indies, intended as a Guide for the young Practitioners in those Countries. By W. J. Evans, Esq. M.R.C.S. —London, 1837. 8vo. pp. 309. 9s.

13. The Nature and Treatment of Dropsy; considered especially in reference to the Diseases of the internal Organs of the Body which most commonly produce it. Parts I. and II. Anasarca and Ascites. To which is added an Appendix, containing a Translation of the Work of Dr. Geronimi, on Dropsy; from the original Italian. By E. J. Seymour, M.D., Physician to St. George's Hospital.—London, 1837. 8vo. pp. 218. 6s.

14. An Extract from Professor Müller, on the Reflex Function of the Spinal Marrow.—Pp. 24. 8vo.

15. A Translation of the Pharmacopœia of the Royal College of Physicians of London, 1836. With Notes and Illustrations. By Richard Phillips, F.R.S. L. & E. &c.—London, 1837. 8vo. pp. 392. 10s. 6d.

16. An Essay on the Influence of Air and Soil as affecting Health, to which was adjudged the Prize offered by the Reverend Thomas Arnold, D.D. to the Students of the Royal School of Medicine at Birmingham. By Alex. Wright, Student.—Birmingham, 1836. 8vo. pp. 56.

17. An Address delivered to the Members of the Royal Medical Society [of Edinburgh], Dec. 16th, 1836. By John H. Bennett, President of the Society. — Edinburgh, 1837. 8vo. pp. 16.

18. Illustrations of the Elementary Forms of Disease. By R. Carswell, Professor of Pathological Anatomy in University College, London, &c. Fasciculus XI. Analogous Tissues.—Fol. 15s.

19. The Jamaica Physical Journal, No. III. and IV. for 1836. Edited by W. Arnold, M.D.—Kingston, 1836.

20. Observations upon the Construction and Use of the Respirator.—London, 1836. 8vo. pp. 12.

21. The Pharmacopœia Collegii Regalis Medicorum Londinensis, translated, with a Commentary, Chemical, Pharmaceutical, and Medicinal. By D. Spillan, M.D.—London, 1837. 12mo. pp. 308.

22. The Works of John Hunter, F.R.S.; with Notes. Edited by J. F. Palmer, Senior Surgeon of St. George's and St. James's Dispensary, &c. In four Volumes, 8vo., and a Volume of Plates in 4to. Vol. I.—London, 1835. 8vo. pp. 643. 17s. 6d.

23. Compendium of Lithotripsy. By Henry Belinaye, Esq. — London, 1837. 8vo. pp. 215. 8s.

24. Observations on the Arrangements connected with the Relief of the Sick Poor, in a Letter to Lord John Russell. By John Yelloly, M.D. F.L.S.—London, 1837. 8vo. pp. 43.

25. The Report of the Poor-Law Committee appointed by the Provincial Medical and Surgical Association. Second Edition, with an Appendix.—London, 1837. 8vo. pp. 89. 2s. 6d.

26. A Literal Interlineal Translation of the first Four Books of Celsus de Medicina, with the "Ordo" and Text, &c. By Robert Venables, A.M. M.B. &c. Second Edition, greatly enlarged.—London, 1837. 8vo. pp. 303. 10s. 6d.

27. Conspectus Medicinæ Theoreticæ. Auctore Jacobo Gregory, M.D. &c. Pars prima.—London, 1837. 12mo. pp. 84.

28. Second Annual Report of the Poor-Law Commissioners for England and Wales.—London, 1836. 8vo. pp. 640. 6s.

29. Outlines of Human Physiology. By Herbert Mayo, F.R.S. &c. Fourth Edition. —London, 1837. 8vo. pp. 334. 18s.

30. Aretæus of the Causes and Signs of Acute and Chronic Disease. Translated from the Greek, by T. F. Reynolds, M.B. F.L.S. &c.—London, 1837. 8vo. pp. 157.

31. A Practical Essay on the History and Treatment of Beriberi. By Assistant Surgeon John Grant Malcomson, Madras Medical Establishment.—Madras, 1835. 8vo. pp. 343.

32. Observations on some Forms of Rheumatism prevailing in India. By Assistant Surgeon John Grant Malcomson, Madras Medical Establishment.—Madras, 1835. 8vo. pp. 98.

33. Practical Observations on the Venereal Disease, and on the Use of Mercury. By Abraham Colles, M.D., Surgeon of Steevens's Hospital, Dublin, &c.—London, 1837. 8vo. pp. 351. 9s.

34. Observations on the Surgical Pathology of the Larynx and Trachea, chiefly with a View to illustrate the Affections of those Organs which may require the Operation of Bronchotomy. By W. H. Porter, A.M., Professor of Surgery in the Royal College of Surgeons in Ireland, &c.—1837. 8vo. pp. 280. 8s.

35. Oration delivered before the Members of the Royal Medical Society of Edinburgh, at the celebration of their Centenary, Feb. 17, 1837. By W. B. Carpenter, senior President of the Society.—Edinburgh, 1837. 8vo. pp. 36.

36. First Principles of Surgery; being an Outline of Inflammation and its Effects. By G. T. Morgan, A.M., Lecturer on Surgery in Aberdeen.—London and Edinburgh, 1837. 8vo. pp. 210.

37. A Treatise on Painful and Nervous Diseases, and on a new Mode of Treatment for Diseases of the Eye and Ear. By A. Turnbull, M.D. Third Edition.—London, 1837. 8vo. 6s.

38. Memoranda for Young Practitioners in Midwifery. By Edward Rigby, M.D. &c.—London, 1837. 48mo. pp. 48. 1s.

39. Principles of Homœopathy. By P. Curie, M.D.—London, 1837. 8vo. pp. 190.

40. Medical Statistics; or, the Causes and Effects of the Extension of Human Life. By J. F. Palmer, M.R.C.S. From the Dublin Review.—London, 1837. 8vo. pp. 16.

FOREIGN.

1. Quæstiones Anatomico-Physiologicæ de Corporibus Wolffianis. Diss. Inaug. Ernesto Dieffenbach, auctore. — Turici, 1836. 8vo. pp. 27.

2. Periodico de la Academia de Medicina de Megico. No. I.—V. — Megico, 1836. 8vo. pp. 160.

3. De Blepahooplastice. Diss. Inaug. Med. Auctore, E. O. Peters. — Lipsiæ, 1836. 4to. pp. 43.

4. Chirurgische Kupfertafeln. Von Dr. Robert Froriep. Heft 69-71.—Weimar, 1837. 4to.

5. Das Heimweh und der Selbstmord. Von J. H. G. Schlegel, M.D. II. Th.—Hildburghausen, 1835. 8vo. pp. 427.

6. Dissertatio de Methodo Endermatica. Auctore A. Ahrensen, M.D. — Hauniæ, 1836. 8vo. pp. 267.

7. Commentatio de Delirio Tremente. Auctore O. C. Höegh-Guldberg, M.D. — Hauniæ, 1836. 8vo. pp. 293.

8. Dissertatio de Hydrocephalo Acuto. Auctore J. O. C. Sommerfeldt.—Hauniæ, 1836. 8vo. pp. 203.

9. De Febre Puerperali Maligna. Auctore S. J. Ballin.—Hauniæ, 1836. 8vo. pp. 94.

10. De Fungo Articulari, Dissertatio. Auctore, Michaele Djörup. — Hauniæ, 1836. 8vo. pp. 116.

11. De Vita et Opinionibus Theophrasti Paracelsi, Dissertatio. Auctore A. F. Bremer.—Hauniæ, 1836. 8vo. pp. 191.

12. Meditationes nonnullæ de Cephalatomia seu Perforatione Cranii. Auctore J. C. Müller.—Hauniæ, 1836. 8vo. pp. 151.

13. Tractatus de Connexu inter Nervum Vagum et Accessorium Willisii. Auctore, H. C. B. Bendz.—Hauniæ, 1836. 4to. pp. 60.

14. Zur Praxis der Geburtshülfe. Beobachtungen und Bemerkungen aus der Academischen Entbindungsanstalt zu Göttingen währerd der beiden Iahre 1822 und 1832. Vom Dr. J. F. Osiander, Professor der Medicin zu Gottingen.—Hannover, 1837. 8vo. pp. 143.

15. Traité de la Consomption Pulmonaire, &c. Par James Clark, M.D. &c. Traduit de l'Anglais, par Henri Lebeau, M.D. &c.—Bruxelles, 1836. 8vo. pp. 388.

16. Beobachtungen auf dem gebiete der Pathologie und Pathologischen Anatomie, gesammelt von Dr. J. F. H. Albers, Prof. der Medizin an der Universität zu Bonn. Erster Theil.—Bonn, 1836. 8vo. pp. 204.

17. Annales, Françaises et étrangères, d'Anatomie et de Physiologie. No. I. Jan. 1837.—Paris. 8vo. pp. 80.

LIBRARY OF THE
Orleans Parish Medical Society

### INDEX TO VOL. III.

OF THE

### BRITISH AND FOREIGN MEDICAL REVIEW.

PAGE

Abercrombie, Dr. on diseases of the brain . . . . . 1
Aberdeen, statistics of suicide in . 270
Abscess, spinal, case of . . 222
hepatic, account of . 340
Abscesses, on the mode of opening 124
Abscess, secondary, Dr. Nasse on . 502
Abdomen, on bandaging, in the puerperal state . . . 267
Abortion, Dr. Davis on . . 408
Abdominal viscera, size and weight of 199
Acid, sulphuric, wounds from . 533
poisoning by . 539
Acid, iodic, mode of preparing . 272
Addison, Dr. on fatty degeneration of the liver . . . . . 155
Addison, Dr. his Practice of Physic 452
Ague, on bleeding in the cold stage of 104
Air-pump, Sir James Murray on . 478
Albers, Dr. his pathological observations . . . . . 220
on putrescence of the uterus 246
Almanack, Medical, British . 206
Alcock, Dr. on the fifth nerve and nerve of taste . . 257-9
Aloes, on the use and abuse of . 264
American Medical Journals . 194
America, state of Medicine in 276, 575
Animal functions, pathology of . 19
Amenorrhœa, treatment of . 398
Amputation, statistics of . . 237
Anderson, Mr. his anatomy of the nervous system . . . 491
Aneurism, thoracic, on the diagnosis of 551
of the arteria innominata 266
Anatomy, Dr. Krause's manual of . 198
Anus, artificial, new mode of treating 518
Apoplexy, Dr. Abercrombie on . 318
Anteversion of the uterus . . 404
Anasarca, Dr. Seymour on . 430
Arterial system, on the pathology of 220
on changes in . 220
Arteries, pathology of . . 69
Artery, intercostal, wound of . 539
Artery, brachial, obstruction of . 242
Arsenic, case of poisoning by . 528
Arnold, Dr. his Jamaica Journal . 192

PAGE

Ashwell, Dr. on chlorosis . . 156
Ascites, Dr. Seymour on . . 436
Asphyxia of new-born children, on the 443
Aurists, English, account of . 81

Books received for review . 298, 589
Buchanan, Mr. his case of cancer cured 557
his surgical report . 558
Brain, on the fibrous structure of . 547
ulceration of . . 549
softening of . 312-3
hypertrophy and atrophy of 317
organic diseases of . . 315
Bronchocele, its topographical relations in India . . . 354
Brigham, Dr. on consumption in America . . . 526
Bandage, immoveable, new form of 515
Bones, Mr. South's description of . 491
Bierkowski, Dr. his Polish report . 475
Bostock, Dr. his System of Physiology 477
Bright, Dr. his Practice of Physic 452
Bloodletting in pneumonia, Dr. Jackson on . . . 456
Broussais, Dr. his reputation declining 448
account of his doctrines 388
Blisters to the eyelids in ophthalmia 450
Bouillaud, Dr. on Medical Philosophy 384
Bichat, account of his theory of disease . . . . 385
Bengal, diseases of, Mr. Twining on 329
British Medical Association . 289
Boisseau, Dr. account of . . 297
Belfast, statistics of fever in . 268
Blood, self-moving power of . 258
diseased, saline medicines in 263
loss of, Dr. Nasse on . 221
Bruit de soufflet, mechanism of . 257
Belladonna, on the fecula of . 229
Bladder, on neuralgia of . . 231
Botany, Himalayan, Mr. Royle on . 205
Bigelow, Dr. on self-limited diseases 201
Barlow, Dr. on ovarian tumours . 165
Balbirnie, Dr. on diseases of the womb, 175
Bright, Dr. on jaundice . . 157
his Practice of Physic . 452
Burns, treatment of, by cold water . 119

PAGE
Bloodletting in ague, Dr. Mackintosh on, 104
Blood, pathology of . . 66

Colquhoun, Mr. on animal magnetism 1
Carcinoma of the breast, Mr. Mayo on 65
Mr. Buchanan on . 558
Cholera, Asiatic, Dr. Mackintosh on 109
Mr. Parkin on . . 179
epidemic, affecting animals 256
Russian and Dutch accounts of . . . 484
Cooper, Sir A. on the ligature of arteries and nerves . . . 154
Chlorosis, observations on . . 156
Cowan, Dr. on intermittence . 162
Chavasse, Mr. on ergot . . 164
Coulson, Mr. on diseases of the hip-joint, &c. . . . 169
Chest, on deformities of . . ib.
Carbonic acid gas, Mr. Parkin on . 179
Chest, Dr. Phillip on diseases of the 184
Dr. Gerhard on diseases of the ib.
deformities of, Dr. Woillez on 226
Cowan, Dr. on physical diagnosis . 184
Corbyn, Mr. his Indian Journal . 192
Celsus, Mr. Lee's edition of . 204
Cataract, Mr. Stevenson on . 207
Crosse, Mr. his anniversary address 208
Congestion, theory of . . 209
Cachexia Africana, account of . 207
Civiale, M. on neuralgia of the bladder 231
Club-foot, on section of tendo Achillis in . . . . . 233
Cæsarean operation, accidental . 248
Dr. Kilian on . 421
Calculus, statistics of, in Austria . 254
Corrigan, Dr. on bruit de soufflet . 257
Conwell, Mr. on diseases of the liver 329
Cochin leg (Bucnemia), Mr. Twining on . . . . . 353
Carmichael, Mr. on tubercle and cancer, 376
Catheterism in the female . . 423
Children, diseases of, Drs. Evanson and Maunsell on . . 439
on the physical education of, 440
Catheter, nasal, account of . . 449
Craigie, Dr. his Practice of Physic 452
Curling, Mr. his treatise on Tetanus 463
Ciliary motions, Dr. Mayer on . 465
Dr. Siebold on . 502
in the brain . 501
Caldwell, Dr. on physical education 472
Clark, Dr. translation of his treatise on Consumption . . 487
Consumption, Dr. Clark's treatise on 487
Combustion, spontaneous, remarkable case of . . . . . 507
Creosote in pulmonary affections, trial of . . . . . 509
Club-foot treated by gypsum . 514
Copaiba injected into the bladder . 521
Carotid artery, ligature of . 522
Cord, umbilical, reposition of . 523

PAGE
Contraction, uterine, how induced . 529
Consumption, American statistics of 526
Clendinning, Dr. on the motion of the heart . . 541
on the sound in percussion . 542
Cancer, open, case of, cured . 557
Croup, spasmodic . . . 445
Clark, Mr. on the anatomy of the nervous system . . . 203

Diseases, on the danger and duration of . . . . . 563
Drowning, morbid appearances from 564
Denmark, state of Medicine in . 565
Discoveries, scientific, recent, in Germany . . . 582
Digestion, on the chemistry of . 541
Diaphragm, on the height of . 542
Dropsy, Mr. Mayo on . . 60
Dr. Seymour on . . 429
Dr. Muller on . . 540
Delivery, case of, after Episioraphy 524
Dieffenbach, his mode of treating club-foot . . 514
fistula 511
on the cure of prolapsus 512
Dickson, Dr. his fallacy of physic . 469
Dilatation, artificial, of the os uteri 413
Davis, Dr. his System of Midwifery 392
Dysentery in India, account of . 330
chronic, mode of treating, 145, 335
Dropsy after scarlatina, account of . 262
Death from blows, medico-legal case of, 248
Dwellings, unwholesome, death from 252
Deaf and dumb, of the, in Brunswick 255
dissection of a person 227
Drunkenness, mode of curing . 231
Diagnosis of chest diseases, books on 184
Dysmenorrhœa, mechanical, treatment of . . . . . 113
Deafness, nervous, account of . 98
Dunglisson, Dr. on the state of Medicine in America . . 273

Edmonds, Mr. on the rate of mortality 40
Ear, diseases of, Dr. Kramer on . 79
curability of . 85
surgery of, history of the . 80
mode of extracting foreign bodies from . . . . 266
Erysipelas, treatment of, by cold water 117
tonic treatment of . 147
Dr. Jackson's treatment of 456
Ergot, Mr. Chavasse on the effects of 165
Elkington, Mr. on the skeleton . 205
Epilepsy, new mode of treating . 263
Edinburgh, gradations at . . 292
Medical Society of . 583
Evanson, Dr. on the diseases of children . . . . 439
Education, physical, Dr. Caldwell on 472

PAGE
Elder-root, use of, in scrofula . 508
Epistaxis, intermittent, case of . 511
Epulis, Mr. Buchanan on . . 559
Elbow-joint, excision of . 483, 559
Eruptive fevers, Dr. Mackintosh on 107
Expatriated omentum, Dr. Parrish on 362
Excito-motory phenomena, Dr. Hall on 33

Fever, typhus, Dr. Parry on . 548
Dr. Lombard on . 260
Indian, Mr. Twining's account of . . . . 347
statistics of, in Belfast . 268
Fevers, classification of . . 103
Fistulæ, vesico-vaginal, treatment of 427, 511
Fluor albus, use of iodine in . 509
Forceps, obstetric, history of . 417
mode of using 410, 418
Fractures of the thigh, M. Josse on 128
French School of Medicine, account of 384
Fees, medical, in Bavaria . . 279
America . . 282
Fœtus, spontaneous amputation of the limbs of . . . . . 267
Forensic medicine, cases in 218, 271
Fifth nerve, anatomy of . . 257
Fracture of the femur, effects of . 258
Foreign bodies introduced into the stomach . . . . 236
Ferguson, Dr. on cachexia Africana 217
Farr, Mr. his Medical Almanac . 206
on the duration of disease 563

Gangræna senilis, its nature . 121
Gangrene from disease of the heart 557
Guy's Hospital, reports from . 142
Gerhard, Dr. on diseases of the chest 184
Gastroperiodynia, Dr. Wise on . 215
Guislain, Dr. on insanity . . 223
Geronimi, Dr. on dropsy . . 436
Greene, Dr. on the diagnosis of aneurism . . . . . 581
Glottis, scalded, case of . . 562
Guyot, M. on fracture of the femur 238

Hospitals in Denmark, account of . 565
Hydrocele, treatment of . . 559
Hernia, curious cause of . . 562
Harrison, Dr. on pectoriloquy . 554
on the height of the diaphragm . 542
Hope, Dr. on rheumatism . 555
Heart, on the motions and sounds of 541, 547
influence of medicines on . 510
on the structure of . . 506
Hannay, Dr. on ulceration of the brain 549
Hunter, John, notice of his works . 486
Hysteria, nature and treatment of . 401
Hernia, Dr. Parrish on . . 355
empirical cure of . . 359

PAGE
Hepatitis in India, account of . 337
Hydrocephaloid disease, account of 325
Homœopathy . . . 288
Herpes, new mode of treating . 241
Hypertrophy of the heart in children 228
Hernia, on the radical cure of . 234
Hemorrhage, theory of . . 211
Hip-joint, on the disease of the . 169
Hysterical paralysis . . 143
Hamilton, Dr. his observations in midwifery . . . 131
Hyperæmia, account of . - 57
Hemorrhoids, Mr. Mayo on . 76
Haller, his doctrine of irritability . 3
Hawley, Dr. on the self-motion of the blood . . . . 258
Hernia, inguino-interstitial . 237
entero-vaginal . . 396
Hyoscyamus, on the fecula of . 229
Hall, Dr. M. on the nervous system 1
reflex function, 33, 577
Humerus, luxation of, new mode of treating . . . . . 241

Infants, on apoplexy in . . 550
Irritation, constitutional . . 1
Isis Revelata . . . 1
Inflammation, Mr. Mayo on . 59
Professor Naumann on 213
its influence in practice 447
Internal ear, diseases of . . 97
Intermittence, nature of . . 162
Insanity, Mr. Neville on . . 189
on the tonic treatment of 223
Ileus, theory of . . . 75
Iralgia cured by quinine snuff . 230
Inversion of the uterus . . 425
Illusions, spectral, singular case of 261
Infants, peculiarities of . . 440
Influenza, account of . . 455
Intestines, internal strangulation of 495
portion of, excised . 517
Italy, state of Medicine in . 577

Johnstone, Dr. biographical notice of 585
Jeffreys, Mr. his Respirator . 492
Joints, on the excision of . . 482
on the disease of . . 146
Journals, German, account of . 460
Jurisprudence, medical, Dr. Traill on 492
Jackson, Dr. on bloodletting . 456
Jaundice, Dr. Bright's account of . 157
in India . . . 343
Jamaica Journal, account of . 193
Josse, M. on Practical Surgery . 145

Kramer, Dr. on diseases of the ear 79
Krause, Dr. his Manual of Anatomy 198
Knox, Dr. on hepatization of the lungs . . . . 259
Kilian, Prof. his Operative Midwifery, 392, 411

PAGE
Key, Mr. on bunions and ganglia . 151
Kennedy, Dr. on apoplexy in infants 550

Ley, Dr. on laryngismus stridulus . 1
Obituary of . . 586
Lupus, on the treatment of . . 556
Liston, Mr. on Nævus . . 557
Lombard, Dr. on the Heart 510
on typhus fever . 260
Louis, M. his treatise on Bloodletting 456
Leeches to the cervix uteri . 428
Leucorrhœa, treatment of . . 400
Liver, morbid anatomy of . . 336
diseases of . . . 329
fatty degeneration of . 155
London College of Surgeons . 292
University, charter of . 293
Larynx, a pebble in . . 265
Lithotomy, mode of practising . 266
Lungs, hepatization of . . 259
Lee, Mr. on mineral waters . 202
Literary men, disorders of . 197
Luxations, spontaneous . . 129
Life, on its admeasurement . 42

Mayo, Mr. his human pathology . 1
Man, observations on . . 40
Mortality, rate of, in different climes 49
Membrana tympani, diseases of . 90
Middle ear, diseases of . 97
Mackintosh, his practice of physic 101
Measles, Dr. Mackintosh on . 108
Medical study, Dr. Symonds on . 201
Magrath, Mr. on oxyde of bismuth 217
Milk-sickness, observations on . 218
Muscles, inflammmation of . 227
Mind, influence of on body . 264
Medicines, comparative use of . 269
price of in America . 282
Man, physical history of . 365
Midwifery, Drs. Davis and Kilian on 392
Menses, cessation of, effect on health 397
Moles, remarks on . . 403
Membranes, on the rupture of in labour 414
Maunsell, Dr. on children's diseases 439
Mouth, gangrene of in infants . 444
Mûhry, Dr. on the state of medicine in France, &c. . . 447
Medicine, state of in England, France, &c. . . 447
Italy . . 575
Denmark . . 565
America . . 575
practice of, Dr. Craigie's 652
Drs. Bright and Addison . ib.
Mayer, Dr. on ciliary motions . 465
Monads, Dr. Mayer's doctrine of . 467
Murray, Sir J. on the air-pump . 478
Manganese, poisonous effects of 452
Macleod, Dr. his report from St. George's . . . ib.

PAGE
Macnish, Dr. death of . . 586
Mateer, Dr. on dropsy . . 548

Nævus, new mode of treating . 557
Nervous system, physiology of . 1
Nervous system, illustrations of . 489
pathology of . 301
Nakkra, a disease of Bengal . 353
Newport, Mr. his discoveries . 291
Neuralgia, diagnosis and treatment of 262, 263
Nævi, treatment of . . 265
Nasse, Dr. on loss of blood . 221
secondary abscess . 502
Naumann, Dr. on congestion, &c. . 209
Nosology, on Botanical . . 452
Nervous system, anatomy of . 203
Neville, Mr. on insanity . . 189
Newnham, Mr. on literary diseases 197
Nieuwenhuys, Dr. on diseases of Amsterdam . . . 161
Nerves, on the diseases of . 20
Nervous system, Dr. Hall on . 1
comparative anatomy of 491

Œsophagus, on diseases of . 72
Organic functions, pathology of . 2
Ovary, enlargement of 165, 138
Osiander, Dr. on the signs of pregnancy 247
Onychia maligna, treatment of . 266
Opium, its use in hernia . . 358
Ophthalmia, use of blisters in . 513
Otto, Dr. on the state of medicine in Denmark . . . 565
Ogston, Dr. on death from drowning 564

Poisoning from yew-berries . 564
arsenic . . 528
sulphuric acid . 539
Patella, on fracture of the . 562
Perforation of the stomach, case of 556
Percussion, Drs. Williams and Clendinning on . . 542-3
Perry, Dr. on typhus fever . 548
Plug, use of, in placenta prævia . 525
Puerperal fever, Dr. Bartels on . 525
Pus, distinctive characters of . 530
Pregnancy, extra-uterine case of 522
Purkinje, Mr. on ciliary motions . 501
Physic, fallacy of the art of . 469
Physiology, Dr. Bostock's system of 477
Perforation, obstetrical . . 420
Perinæum, rupture of . . 422
Prolapsus uteri, treatment of . 424
Pelvis, admeasurement of . 393
Pregnancy, signs of . 140-247-405
Parrish, Dr. on hernia . . 355
Prichard, Dr. on the physical history of man . . . . 365
Philosophy medical, M. Bouillaud on 384
Pinel, his nosography . . 387
Provincial medical association . 290

70

PAGE
Pork, poisonous effects of . 272
Penis, amputation of . 266
Pelvis, state of in luxation . 240
Pregnancy, tubal, with rupture . 245
Pathological observations by Albers 220
Plethora, theory of . 211
Parkin, Mr. on cholera, &c. . 179
Phillipp on diseases of the chest 184
Provincial Association, transactions of 159
Prolapsus of the uterus . 132
Pessaries, their use in prolapsus . 134
Polypus of the uterus . 135
Porrigo of the head . 143
Pathology, human, Mr. Mayo's . 55
Pectoriloquy, Dr. Harrison on . 554
Palmer, Mr. his edition of Hunter's works . 486
Prize essays for 1837-8 . 287
Paralysis, Dr. Abercrombie on . 320

Quetelet, M. his essay on man . 40
Quickening, on the signs of . 405

Review, books received for 298-589
Rheumatism, treatment of . 555
Rosas, Prof. his ophthalmic report 517
Respirator, account of . 492
Rigby, Dr. his obstetrical memoranda 494
Rokitanski, Dr. on strangulation of the bowels . 495
Retroversion of the uterus . 427
Royal Society, prize medals of 291
Royle, Mr. his botany . 205
Roots, Dr. on porrigo . 143
Reflex function of the spinal cord 33, 577

Spinal cord, pathology of . 29
Sympathy, phenomena of . 34
Systems, medical, of Germany . 451
Shoulder-joint, excision of . 482
Swan, Mr. his illustrations of anatomy 489
South, Mr. his description of the bones 491
Syme, Mr. his principles of surgery 481
Surgery, principles of . ib.
Sims, Dr. on diseases of the brain 1
Speculum, vaginal, use of . 175
for the ear . 88
Syringing the ear, mode of . 89
Scarlet fever, Dr. Mackintosh on 108
Small pox, Dr. Mackintosh on . 109
Saline injections in cholera . 112
St. Thomas's Hospital, reports from 142
Self-limited diseases . 200
Symonds, Dr. on medical study . 201
Stevenson, Mr. on cataract . 207
Solaneæ, on the extracts of . 229
Stramonium, on the fecula of . ib.
Stuttering from worms, case of . 231
Skin diseases of, sulphuret of lime in 242
Stark, Dr. on dropsy after scarlatina 262
Spasm of the muscles of the neck 263
St. John Long, his liniment . 264

PAGE
Suicide, statistics of . 270
Softening of the brain, Dr. Sims on . 313
Sims, Dr. on softening of the brain . ib.
Spleen, diseases of, in India . 344
Seymour, Dr. his treatise on dropsy . 429
Siebold, on ciliary motions . 502
Salivation, use of iodine in . 511
Statice armeria, a diuretic . ib.
Staphyloma, dissection of a case of . 521
Stomach, perforation of . 556
Syphilis, hydriodate of potash in . 561
St. George's Hospital, report of . 552
Skeleton, Mr. Elkington on the . 205

Turner, Dr. biographical notice of . 583
Travers, Mr. on hydrocele . 560
Thomson, Dr. on the chemistry of digestion . 541
Traill, Dr. his medical jurisprudence 492
Tar water in pulmonary affections . 509
Turning, in midwifery . 416
Tuberculous diseases, Mr. Carmichael on . 376
Twining, Mr. on the diseases of Bengal 329
Tracheotomy, cases requiring . 265
Tic douloureux, case of, cured . 230
Thyroid gland, Sir A. Cooper on . 153
Transactions of the Provincial Association . 159
Tyrrell, Mr. on diseases of the joints 146
Tympanum, perforation of . 92
Transactions of Med. Chir. Society 1
Travers, Mr. on constitutional irritation 1
Tetanus, Mr. Curling on . 463
pathology of . 464
Translation of medical works, remarks on . 488

Urethra, inflammation of, peculiar . 239
stricture of, new treatment of 242
Umbilical cord, injections into . 244
Uterus, putrescency of . 246
United States, state of medicine in . 273
Ure, Dr. on the treatment of lupus 556
Uterus, extirpation of . 561

Vagina, laceration of . 562
Velpeau, M. on blisters to the eyelids 513
Venereal diseases, local treatment of 516
Version, spontaneous, of the fœtus . 243
Vocabulary, medical . 206
Veins, inflammation of . 68

Williams, Dr. on percussion . 543
Watson, Dr. on the sounds of the heart . 547
Wounds from sulphuric acid, case of 533
gunshot, case of . 536
Wright, Mr. on the influence of the air 494
Worms, causing stuttering . 281
Wise, Dr. on gastroperiodynia . 215
Watering places, continental . 202

PAGE
Water, cold, its use in surgery . 115
Wounds, lacerated, treatment of . 120
on the mode of dressing . 126
Williams, Dr. his Elements of Medicine . . . . 452
Womb, on the diseases of . 175

Yew-berries, poisoning by . 564
Yellow fever, treatment of . 179
on oxide of bismuth in 217

END OF VOL III.

